# Polio revisited: reviving knowledge and skills to meet the challenge of resurgence

**DOI:** 10.1007/s11832-015-0678-4

**Published:** 2015-09-11

**Authors:** Benjamin Joseph, Hugh Watts

**Affiliations:** Aster Medcity, Kochi, Kerala India; Shriners Hospital for Children, Los Angeles, CA USA; 18 HIG HUDCO Colony, Manipal, Karnataka 576104 India

**Keywords:** Poliomyelitis, Resurgence, Surgical decision-making, Bracing, Paralytic deformity

## Abstract

**Purpose:**

To date, polio has not been eradicated and there appears to be a resurgence of the disease. Hence, there is a need to revive decision-making skills to treat the effects of polio.

**Methods:**

Here, we outline the aspects of treatment of paralysis following polio based on the literature and personal experience of the authors. The surgical treatment of the lower and upper extremities and the spine have been reviewed. The scope of bracing of the lower limb has been defined.

**Results:**

The effects of polio can be mitigated by judicious correction of deformities, restoration of muscle balance, stabilising unstable joints and compensating for limb length inequality.

**Conclusions:**

As polio has not been eradicated and there is a risk of resurgence of the disease, paediatric orthopaedic surgeons need to be prepared to deal with fresh cases of polio. Revival of old techniques for managing the effects of paralysis following polio is needed.

## Introduction

The dream of eradicating polio globally by 2000 ad has not been fulfilled. On the contrary, fresh outbreaks of polio have been reported in this century not just from parts of the developing world but even from countries previously declared polio-free [1–5]. Immunization programmes have been thwarted by war, terrorism and failure of governments to sustain universal immunization targets [2, 6–8]. Polio is still endemic in Pakistan and Afghanistan and fresh cases of paralytic polio continue to be reported from these countries [9]. Nigeria is only just approaching the target with 12 months having elapsed since the last reported case due to wild poliovirus. These trends have made the World Health Organisation declare the situation a ‘public health emergency’ [10].

While this is a serious public health problem what is it to us as paediatric orthopaedic surgeons?

As we may encounter children with residual effects of polio either in our own country or while offering humanitarian service in regions of the world with limited resources, we need to know if we are adequately prepared to deal effectively with children with residual paralysis of polio. Our impression is that we may not be. The vast majority of paediatric orthopaedic surgeons with a reasonable experience in dealing with polio are now in the sixth, seventh or eighth decade of life; younger surgeons have seldom, if ever, dealt with a case of polio. Current editions of several standard textbooks on operative orthopaedics do not include sections on polio. Curricula of general orthopaedic and specialized paediatric orthopaedic training do not include polio. Consequently, decision-making skills and surgical skills may be found wanting. Due to the need to revise our knowledge and revive our decision-making skills to deal with the resurgence of this disease, we decided to review the management of polio.

## General principles of orthopaedic treatment of polio

Paralytic polio passes through three stages—paralysis, recovery, and residual paralysis. The paediatric orthopaedic surgeon may be called upon to treat a child in any of these stages of the disease.

### Treatment in the stage of acute paralysis

Splinting of the paralyzed limb in the functional position is essential to prevent postural deformities from developing.

### Treatment during the stage of recovery

Once recovery begins, attempts must be made to get the child to stand and walk with an appropriate orthosis based on the pattern of paralysis. With progressive recovery, the extent of bracing may be reduced.

### Treatment of permanent residual paralysis

The pattern of paralysis in polio is characteristically asymmetric and muscles that are affected most frequently are those that have all their anterior horn cells in a small localized area in the spinal cord (e.g., tibialis anterior, quadriceps femoris, deltoid, opponens pollicis). The consequences of paralysis of muscles are motor weakness, muscle imbalance, joint instability and shortening of the limb. Muscle imbalance, in turn, can lead to deformity. Each of these needs to be evaluated by careful physical examination and addressed as shown in Table 1.

**Table 1:** Consequences of muscle paralysis and the treatment options

Consequence of muscle paralysis	Options for intervention
Motor weakness	Tendon transfer if muscle of adequate power (Grade V on the MRC scale) is available
	Bracing if muscle of adequate power is not available
Muscle imbalance	Tendon transfer from stronger side of the joint to the weaker side of the joint if muscle of adequate power is available
	Weaken muscles on stronger side of the joint if muscle of adequate power is not available for transfer
Instability of joint	Tendon transfer if: (a) Muscle of adequate muscle power available for transfer, (b) Instability is unidirectional (possibly bi-directional around ankle and foot)
	Osteotomy to alter the biomechanics and restore stability (e.g., shifting the axis of movement of the joint)
	Bracing if tendon transfer or osteotomy is not feasible
	Arthrodesis (appropriate only for spine, shoulder, wrist and foot)
Deformity	Soft tissue contracture release (release of tendons, fascia, joint capsule)
	Osteotomy for residual deformity after soft tissue release
	Ignore if deformity contributes to stability (e.g., mild genu recurvatum or mild equinus in child with quadriceps paralysis)
Shortening of lower limb	Lengthening of short lower limb
	Growth arrest of long lower limb
	Compensate with shoe lift (especially if orthosis is required for shorter limb)
	Ignore shortening in the upper limb and if <2 cm in the lower limb

Specific treatment will vary from region to region as outlined later in this review.

## Treatment of the hip in polio

### Muscle paralysis

Occasionally, the hip may be flail due to paralysis of all its muscles; however, more frequently some muscles are spared. Paralysis of the hip abductors results in a Trendelenburg gait which is both unsightly and grossly energy inefficient. Tendon transfers to replace the function of these powerful antigravity muscles may improve the power of abduction by one or possibly two grades on the Medical Research Council (MRC) scale but seldom restore it to normality. Occasionally, the transfer may only serve as a tenodesis without appreciable gain in motor power. Nevertheless, the Trendelenburg lurch may be minimized with iliopsoas and external oblique tendon transfers or a free gracilis transfer [11–14]. Paralysis of the gluteal maximus is also disabling with a characteristic lurch where the lumbar spine arches backwards during the stance phase of gait to compensate for the loss of hip extension power. Erector spinae transfer can minimize the lurch [15, 16].

### Deformities

**Fig. 1:**
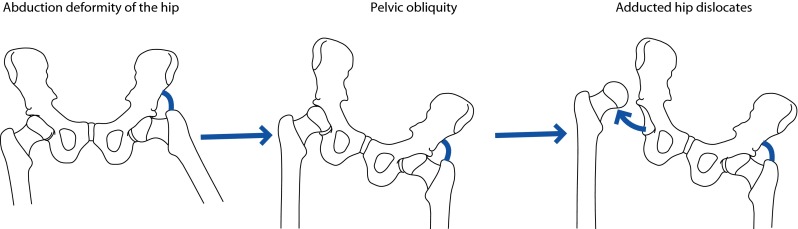
Pelvic obliquity due to an abduction contracture of one hip can result in dislocation of the opposite hip

Deformities of the hip commonly seen in polio are flexion, abduction and external rotation (often in combination); adduction and internal rotation are less frequent. Abduction or adduction deformities will cause pelvic obliquity and a compensatory lumbar scoliosis and the pelvic obliquity can predispose to hip dislocation (Fig. 1). To begin with, the deformities are due to soft tissue contractures, although in long-standing cases adaptive bony changes may supervene. Soft tissue release will correct all but the severe deformities; osteotomies of the proximal femur may be needed to correct any residual deformities that remain after the soft tissue release. Careful muscle power testing prior to soft tissue release is of paramount importance while dealing with an abduction deformity. If the hip abductors are paralyzed, retaining a mild degree of a fixed abduction deformity can prevent a Trendelenburg gait (i.e., under-correction is desirable if there is hip abductor weakness).

### Instability

Hip subluxation or dislocation may occur in children with polio. Muscle imbalance is the prime factor that leads to hip instability with bony adaptive changes such as femoral anteversion, coxa valga and acetabular dysplasia contributing to the problem. All of these need to be addressed to restore hip stability [17–21].

The problems related to the hip and their consequences are summarised in Table 2.

**Table 2:** Patterns, consequences and treatment options of hip problems in polio

Problems	Common patterns	Consequences	Treatment options
Motor weakness	Hip abductor weakness	Trendelenburg gait	Iliopsoas transfer External oblique transfer
	Hip extensor weakness	Gluteus maximus lurch	Erector spinae transfer
Muscle imbalance	Hip flexor stronger than hip extensor	Flexion deformity of the hip resulting in knee flexion with instability in stance if the quadriceps is also weak	Hip flexor release
	Hip flexor and adductor stronger than hip abductor and extensor	Flexion/addiction deformity and tendency for paralytic subluxation and dislocation of the hip	Hip flexor/adductor release OR Iliopsoas transfer after contracture release
Deformity	Flexion, abduction, external rotation	Pelvic obliquity	Soft tissue release (sartorius, tensor fascia lata, rectus femoris, anterior fibres of gluteus medius and minimus, iliopsoas, anterior capsule of hip) Proximal femoral osteotomy
Instability	Trendelenburg gait without hip subluxation due to abductor weakness	Instability during stance phase of gait	Iliopsoas or external oblique tendon transfer
	Paralytic subluxation or dislocation due to muscle imbalance	Joint instability	Restore muscle balance with iliopsoas tendon transfer and correct coxa valga, femoral anteversion and acetabular dysplasia

## Treatment of the knee in polio

### Muscle paralysis and knee instability

**Fig. 2:**
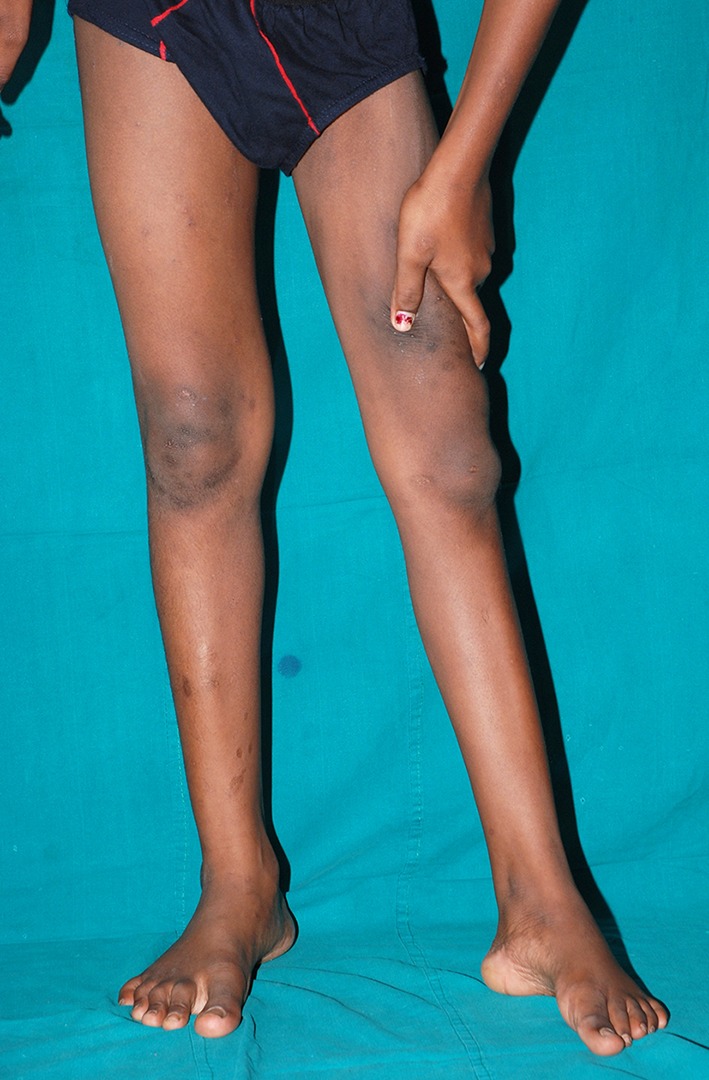
Hand-to-thigh gait adopted by a boy who has paralysis of his left quadriceps femoris muscle

Quadriceps paralysis is more frequent than paralysis of the hamstrings and quadriceps paralysis is far more incapacitating. Knee instability in the stance phase of gait occurs when the quadriceps power is less than Grade II on the MRC scale. Children with Grade III power may walk well on an even surface on level ground but often experience instability while walking on uneven ground or while negotiating slopes or stairs. These children need to support the thigh with the hand to prevent the knee from buckling; they adopt the characteristic ‘hand-on-thigh’ gait (Fig. 2), which requires adequate triceps strength while tying up the hand for other use.

Active knee extension can be restored by performing a transfer of the biceps femoris and the semitendinosus to the front of the knee [22, 23]. However, not all children are candidates for the transfer; the hip extensor power and ankle plantarflexor power should be normal and there should be no flexion or recurvatum deformities of the knee [24].

In children who do not fulfill these criteria for a hamstring transfer, stability of the knee can be restored by the use of a floor-reaction orthosis or a knee−ankle–foot orthosis (see section on the scope of bracing). In older patients, the orthosis can be abandoned by performing a supracondylar extension osteotomy to create 10−15° of genu recurvatum. This operation is performed closer to skeletal maturity (i.e., >10 years of age) as remodeling of the osteotomy will occur with recurrence of stance phase instability in the young child.

### Deformities

**Fig. 3:**
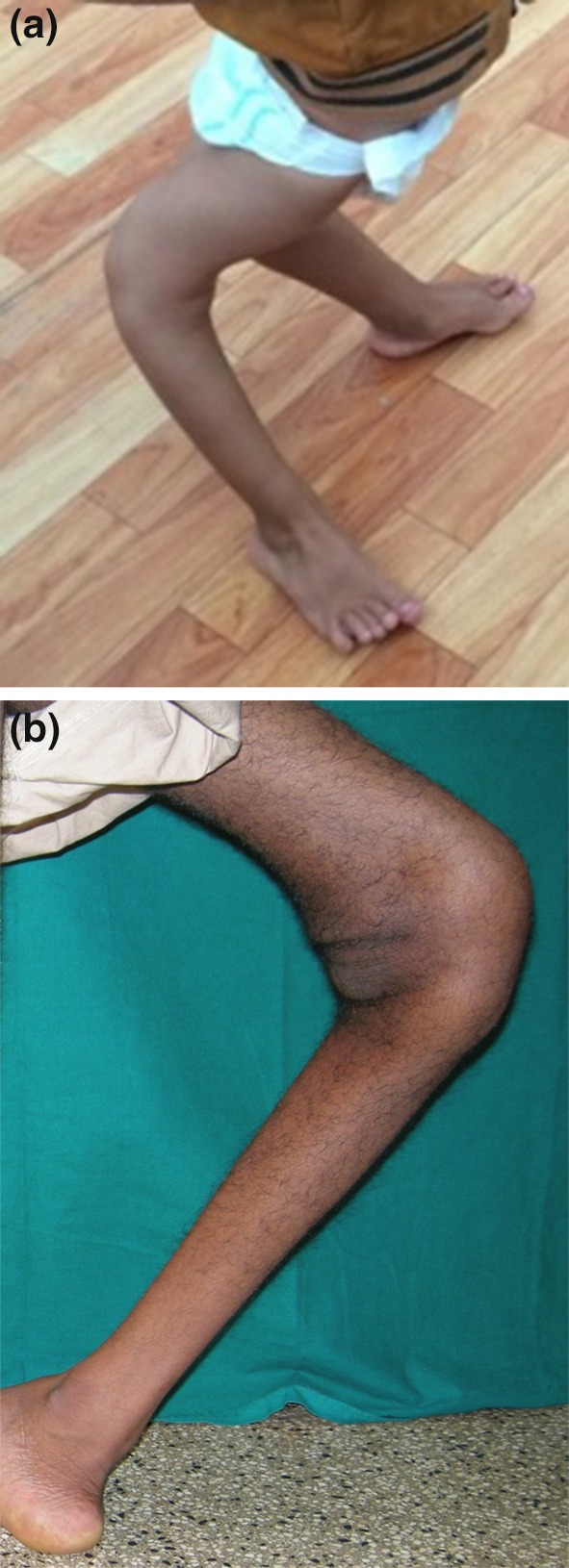
A young boy (**a**) and an adolescent (**b**) with severe genu recurvatum

Flexion deformity and genu recurvatum are commonly seen and may be very severe (Fig. 3).

A mild degree of genu recurvatum or a mild equinus deformity is beneficial in children with quadriceps paralysis as these deformities will stabilize the knee in the stance phase; it is important that these mild deformities are not corrected if there is weakness in the quadriceps.

**Fig. 4:**
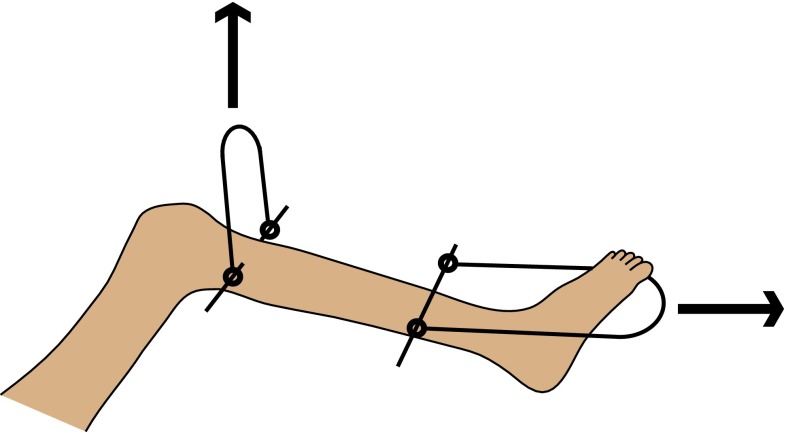
Skeletal traction for severe fixed flexion deformity of the knee should include anterior traction on the proximal tibia to prevent posterior subluxation of the knee along with longitudinal traction to correct the flexion deformity

Mild degrees of flexion deformity may be corrected by serial casting while a moderate deformity will need release of the contacted hamstrings. Severe flexion deformity may be corrected by a combination of hamstring release and supracondylar extension osteotomy or soft tissue release followed by skeletal traction. A precaution that should be taken while correcting severe flexion deformity by traction is to prevent posterior subluxation of the knee by applying anteriorly directed traction on the proximal tibia in addition to the longitudinal traction for deformity correction (Fig. 4). Recurvatum >15° must be corrected by bracing or a supracondylar flexion osteotomy.

External rotation deformity of the tibia can result from a tight tensor fascia lata; if severe, it obviates the ability of ankle plantar flexion to aid in extending a weak knee. Early release of the ilio-tibial band just above the knee (Yount release) may significantly improve moderate degrees of external rotation deformity of the tibia. If the rotation is severe, a proximal internal rotation osteotomy of the tibia may be necessary.

## Treatment of the foot and ankle in polio

### Muscle paralysis

The foot and ankle are often affected in polio with varying degrees and patterns of paralysis ranging from partial paralysis of a single muscle to a flail foot with complete paralysis of all muscles.

### Deformities

**Fig. 5:**
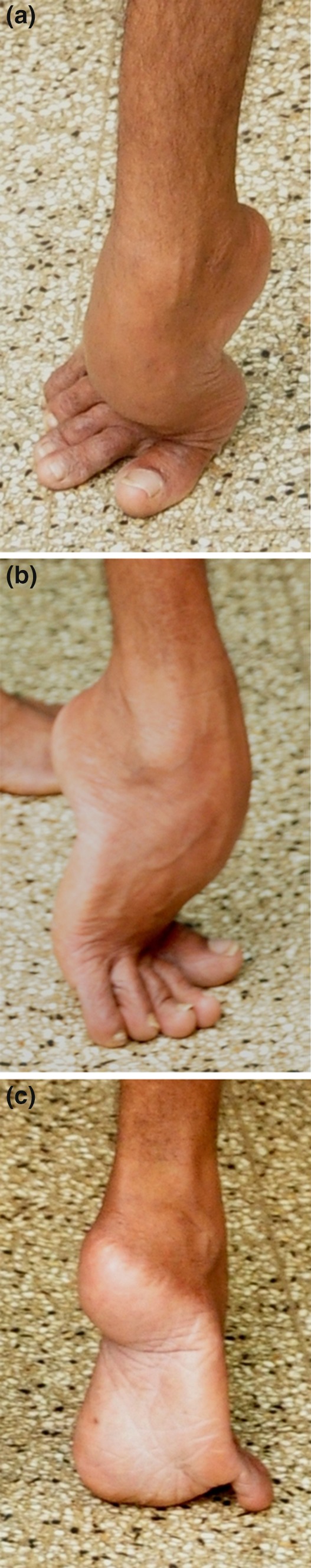
An equino-cavo-varus deformity in an adolescent with polio. The equinus (**a**), cavus (**b**) and the hind foot varus (**c**) components of the deformity are clearly seen

**Fig. 6:**
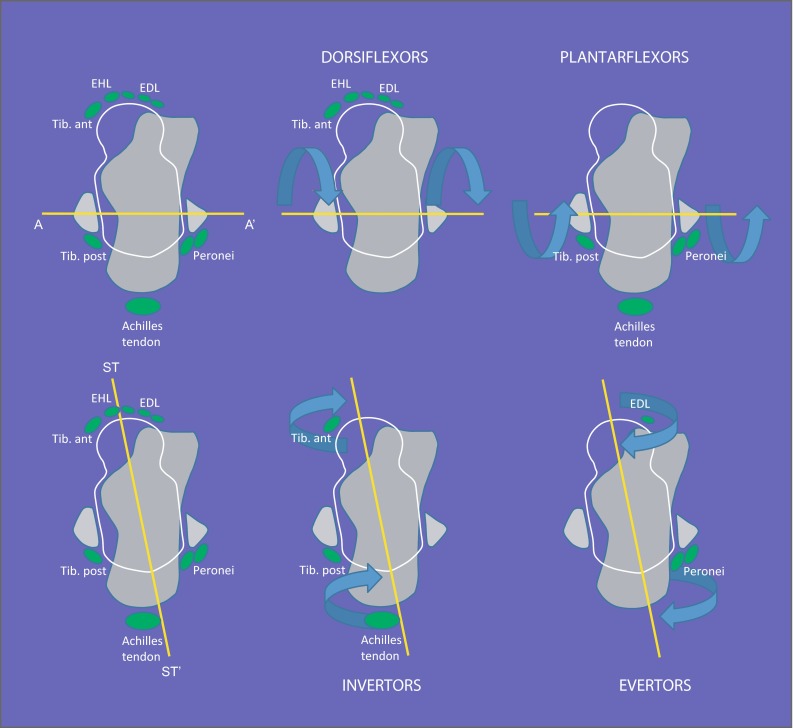
The location of tendons in relation to axes of the ankle joint (*AA′*) and the subtalar joint (*STST′*) are shown. All tendons located anterior to the axis of the ankle are ankle dorsiflexors (*top-middle*) while all tendons located posterior to the axis of the ankle are plantarflexors (*top-right*). All tendons located medial to the subtalar axis are invertors (*bottom-middle*) while all tendons located lateral to the subtalar axis are evertors (*bottom-right*). The greater the perpendicular distance of these tendons from the respective axis, the greater is their force moment

**Fig. 7:**
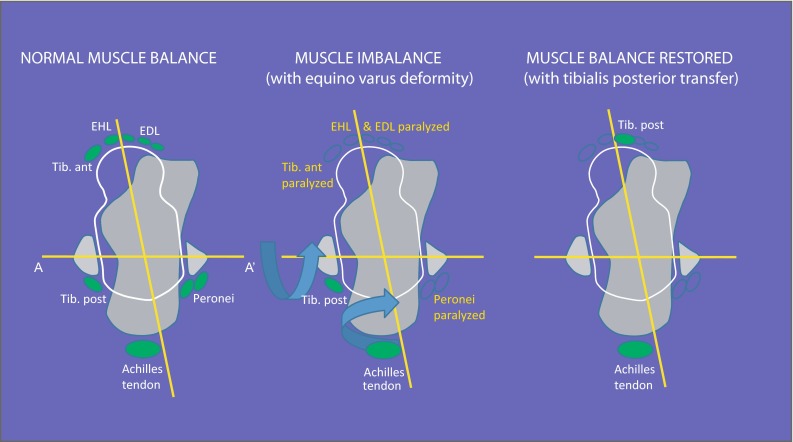
Loss of muscle balance across the joint axis can result in a deformity. Restoration of muscle balance by an appropriate tendon transfer can correct the deformity

**Fig. 8:**
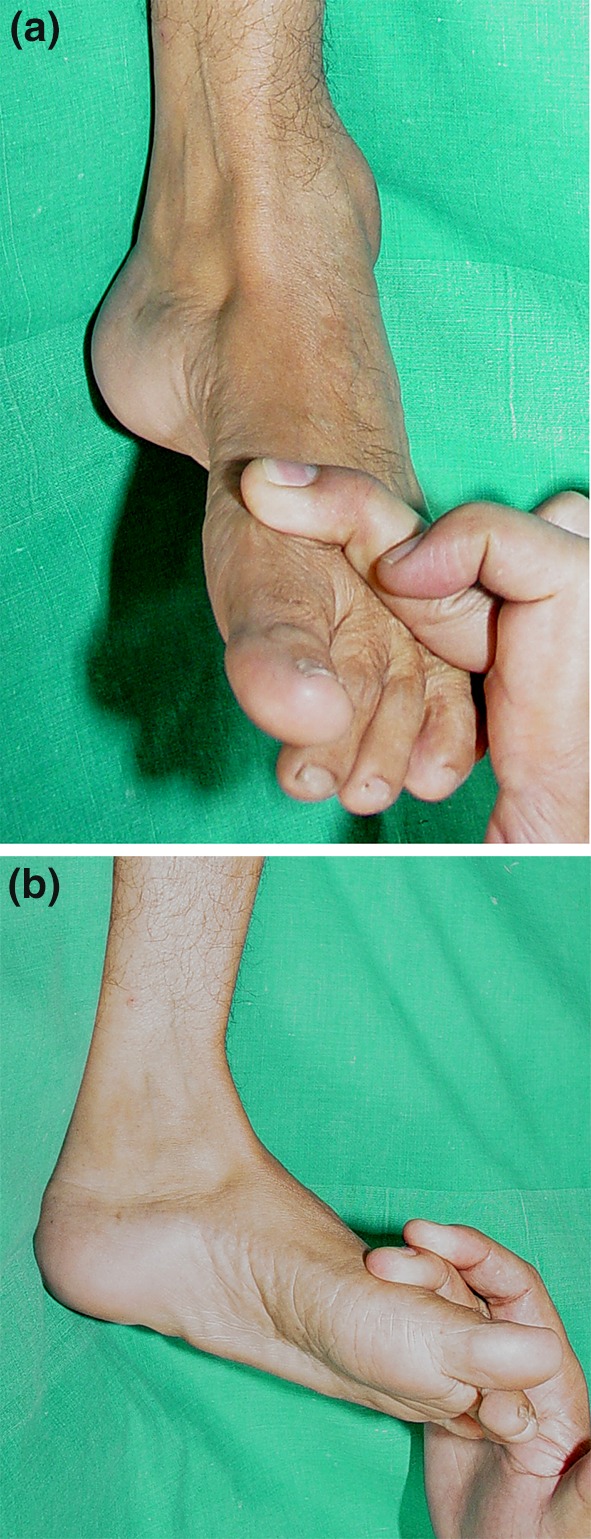
Manual muscle testing of power of ankle dorsiflexors (**a**) and the invertors (**b**)

A wide spectrum of hindfoot and ankle deformities may occur, including equinus, calcaneus, varus, valgus, pes planus and cavus and a combination of these (e.g., equino-varus, equino-valgus, calcaneo-varus, calcaneo-valgus, calcaneo-cavo-valgus, equino-cavo-varus; Fig. 5). A clear understanding of the force moments of muscles acting on the ankle and subtalar joints will facilitate the choice of the appropriate treatment (Fig. 6). The pattern of deformities is governed by the pattern of paralysis and the resultant imbalance that develops across the axes of the ankle and subtalar joints. The tendon to be transferred and the point of attachment following the transfer should be such that muscle balance across both these axes is restored (Fig. 7). Careful estimation of the muscle power by manual muscle testing is extremely important in planning treatment and the power of each muscle must be documented before contemplating a tendon transfer (Fig. 8a, b) as the muscle selected for transfer must have normal or near normal power (Grade IV or V) for the transfer to be effective.

**Fig. 9:**
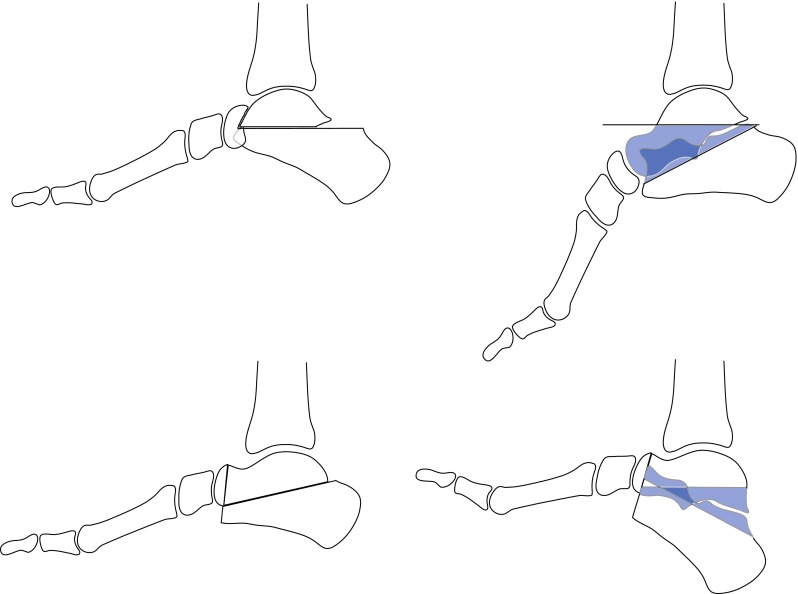
Wedges resected from the talus and calcaneum during triple fusion for equino-cavus (*above*) and calcaneus (*below*)

**Fig. 10:**
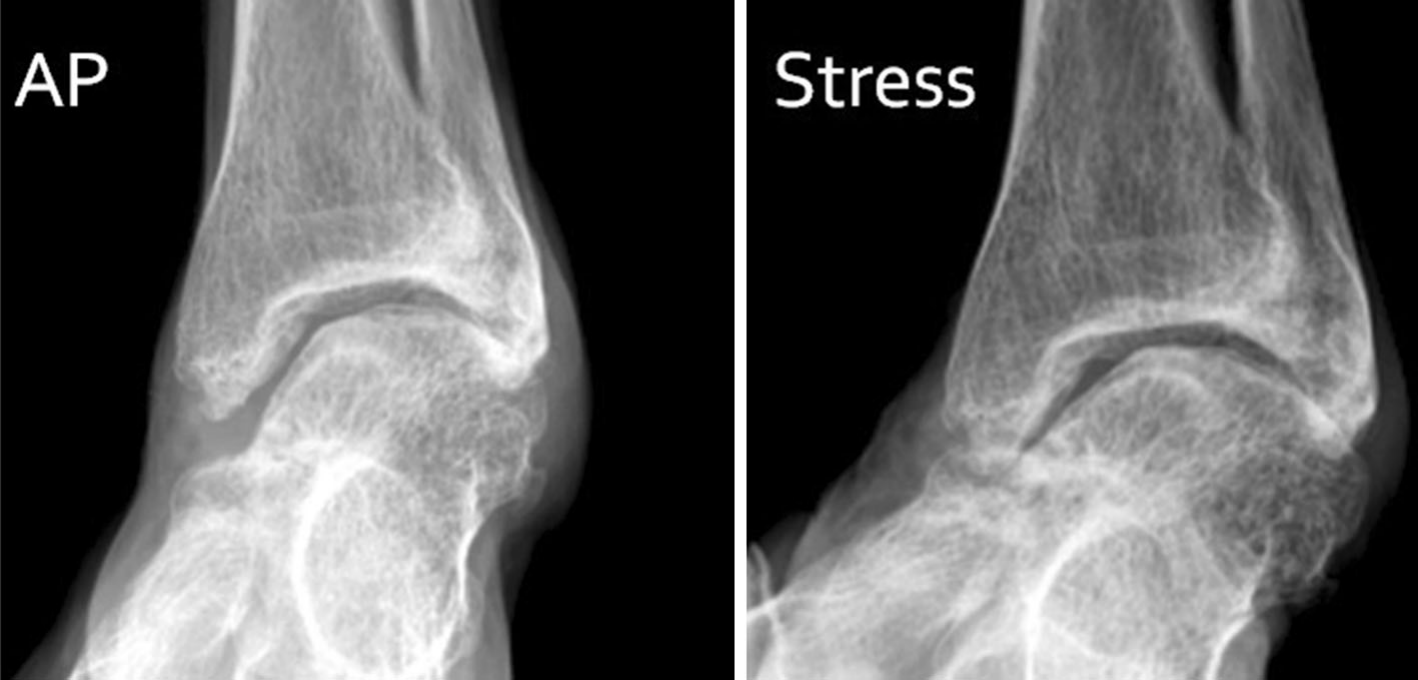
Varus deformity of the ankle, acquired ball-and-socket ankle, degenerative arthritis and varus instability developed 25 years after a triple fusion was performed to correct a varus deformity at the subtalar level in an adolescent. The underlying muscle imbalance was not corrected at the time of surgery

Established deformities of the foot may be corrected by releasing or lengthening contracted tendons in the younger child but bony surgery may be needed in older children. Resection of wedges of bone from the tarsus often facilitates the correction of rigid deformities; the wedge resection should be extra-articular whenever possible. Wedges that include the articular surfaces of joints may be excised when arthrodesis of the joint is planned as part of the strategy of deformity correction. Variations of the triple arthrodesis which entails fusion of the talo-calcaneal (sub-talar), talo-navicular and calcaneo-cuboid joints can correct a variety of foot deformities in older children and adolescents [25–27]. The location and orientation of the wedge will vary with different deformities (Fig. 9). It is extremely important to restore muscle balance at the time of performing a triple fusion; failure to do so will result in late secondary deformities at the ankle as the unbalanced forces will act on the mobile joint proximal to the level of fusion (Fig. 10).

In the forefoot, dorsal bunion of the first metatarsal may occur if there is isolated weakness of the peroneus longus muscle, or if the peroneus longus has been transferred without a concomitant transfer of the tibialis anterior muscle from the first metatarsal if the latter muscle is functioning.

### Instability

Instability of the ankle occurs when both the dorsiflexors and plantarflexors are paralysed and instability of the subtalar joint occurs when the invertors and evertors are paralysed or when the joint is rendered flail after a tendon transfer performed to improve ankle function. Instability of the subtalar joint that allows the calcaneum to go into valgus can obviate the effect of the ankle plantarflexors to help extend the knee when there is knee extension weakness; however, sub-talar arthrodesis can avoid this.

## Treatment of the upper limb in polio

### Muscle paralysis

The muscle most frequently paralysed is the deltoid and when it is completely paralyzed the rotator cuff muscles are also often paralysed [28]. The elbow flexors or extensors may be paralysed, and the opponens pollicis is the muscle in the hand that is frequently affected.

**Fig. 11:**
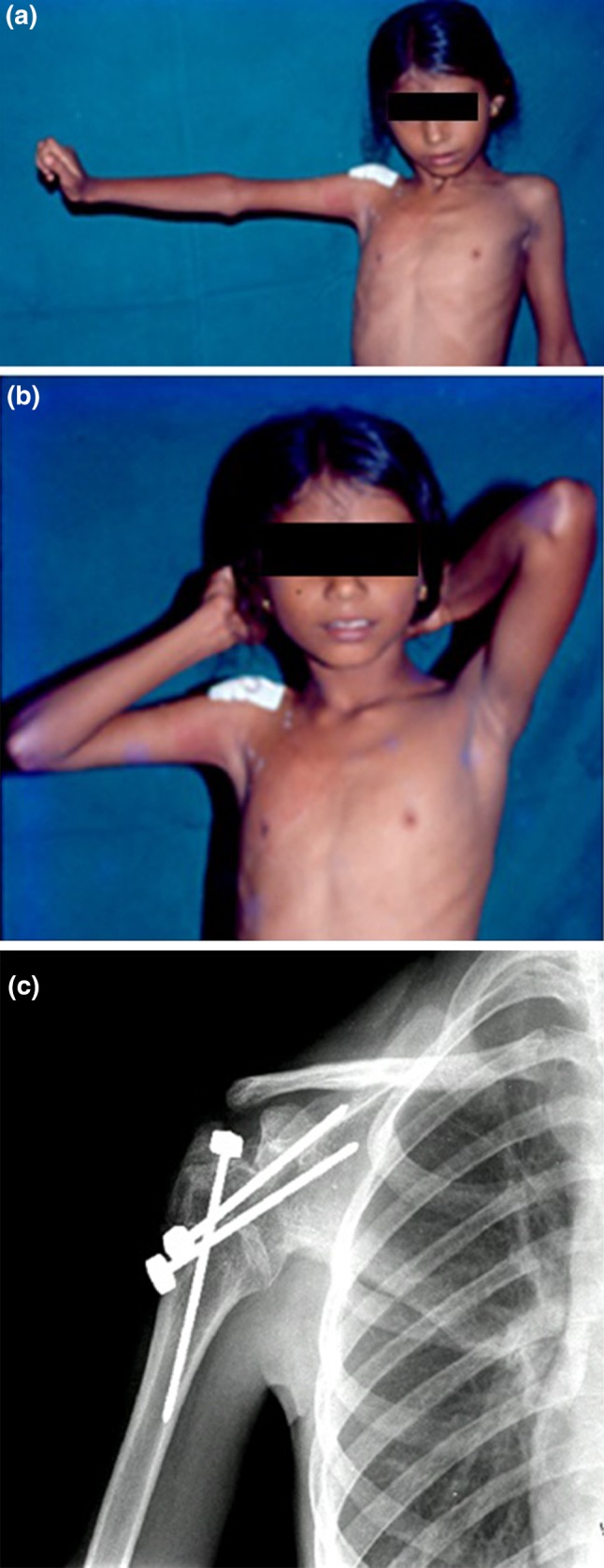
Active abduction of the shoulder (**a**) which enables a girl to tie her hair (**b**) is possible after arthrodesis of a flail shoulder (**c**)

Although tendon transfers have been described for dealing with shoulder paralysis [29, 30] they are not very popular. Shoulder arthrodesis is an option if the scapulo-thoracic muscles are functioning normally; it is recommended in children after the age of 7 years when there is complete paralysis of the deltoid. Significant improvement in function has been noted following this procedure [31–33] (Fig. 11).

It is important to restore strong active elbow flexion when the elbow flexors are weak or paralyzed; the Steindler flexorplasty or triceps transfer to the front of the elbow are useful. The former operation, that entails moving the common flexor origin proximally, may improve weak elbow flexion but will not restore useful active flexion if the biceps brachii and the brachialis are totally paralyzed. Triceps transfer is likely to be more effective in children with completely paralyzed elbow flexors but may lead to the inability to use a crutch or reach back to propel a wheelchair. Consequently, a triceps transfer is contra-indicated in children who are likely to need crutches or a wheelchair.

**Fig. 12:**
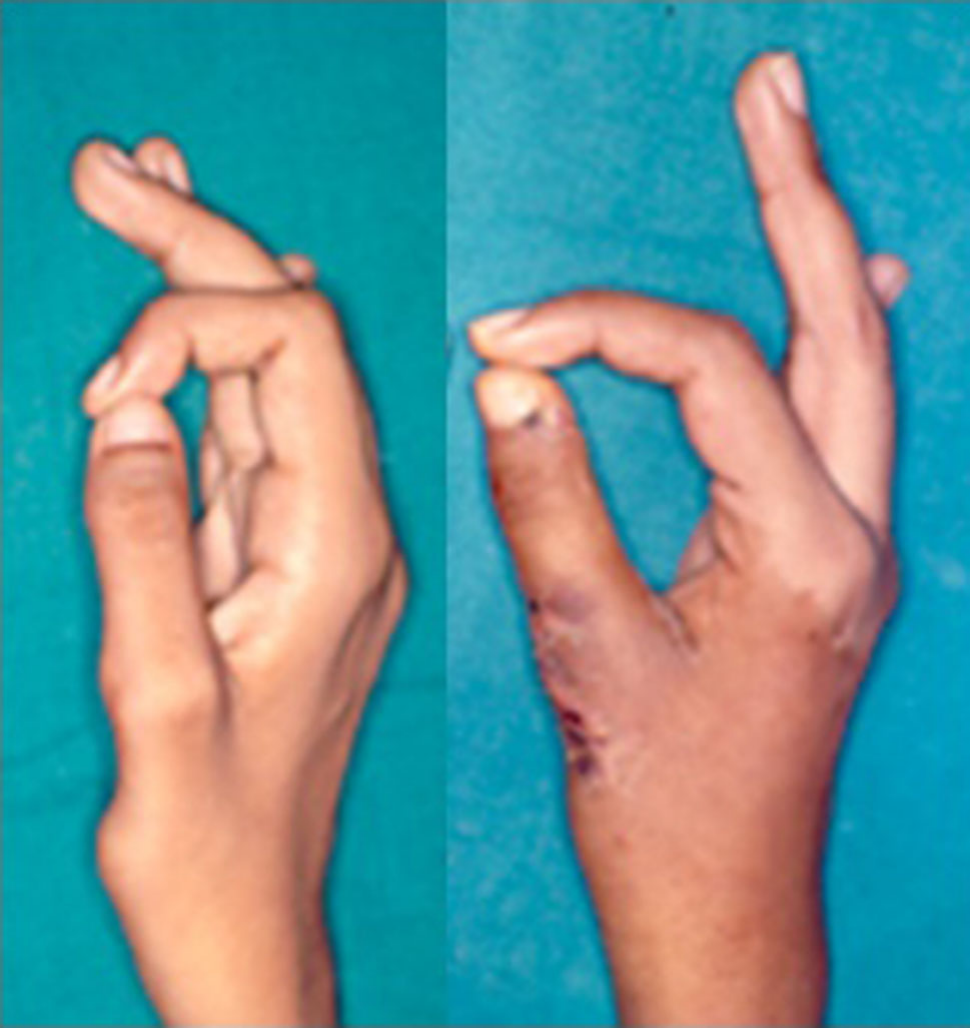
Poor opposition of thumb in a child with paralysis of the opponens pollicis following polio (*left*) and pulp-to-pulp opposition restored after opponensplasty with flexor digitorum superficialis transfer (*right*)

Paralysis of the opponens pollicis with inability to oppose the thumb effectively can be treated with a transfer of the flexor digitorum superficialis of the ring finger (Fig. 12). If the child must use crutches to walk, the force of the crutch handle may stretch out an opponens transfer. A synostosis between the first and second metacarpal bones may be a wiser choice.

## Treatment of the spine in polio

Scoliosis in poliomyelitis is seen in two groups of children:

1. The first are young children who suffer extensive paralysis of the trunk muscles and develop scoliosis very early, commonly within 2 or 3 years of the paralytic episode. The scoliosis in these children tends to be in the thoracic spine and respiratory impairment often ensues. In regions with very limited resources, these children commonly die in early childhood.
2. The second are children in whom scoliosis develops gradually in later childhood. The scoliosis is usually in the lumbar region and the spinal curve may compromise the ability to walk or sit.

### Incidence

Estimates of the incidence of scoliosis following poliomyelitis are often imprecise since they are based on physical examination rather than by X-ray. In the Tajikistan polio outbreak of 2010, 39 of the 360 people personally (HW) evaluated by physical examination had scoliosis (11 %). All but two were <10 years of age at the time they contracted poliomyelitis. Of the children who contracted poliomyelitis <10 years of age, 18 % had trunk involvement; <1 % of those who contracted poliomyelitis >10 years of age demonstrated trunk involvement.

### Problems of management

The management of scoliosis in poliomyelitis is very different from that of idiopathic scoliosis. The alignment and mobility of the spine can influence the ability to walk and, consequently, the spine must not be considered in isolation from the rest of the body.

Loss of lumbar lordosis following spine surgery can be a major impairment to walking, or even sitting, if the hip extensors are weak since there will be no way for the child to lean back sufficiently to get the mass of the trunk posterior to the hip joints.

A supple lumbar spine may be necessary not only for forward movement but also lateral ‘balance’. This may be insignificant in a crutch-free and brace-free child, but it may be catastrophic in a marginal household walker. Fusing the lumbar spine may decrease (or totally prevent) the patient’s ability to walk, whether or not the sacrum is included. Parents not warned of this potential difficulty will be justifiably upset if their child stops walking after a spine fusion.

A severe lumbar curve can be a major difficulty in walking due to the resulting apparent leg length inequality. Additionally, if a child’s curve is very supple, he may need to expend an extra effort to stretch out the spine before the push on the crutch straightens the spine sufficiently to allow clearance during swing phase.

### Examination of scoliosis associated with poliomyelitis

The physical examination of a child with scoliosis following poliomyelitis should not focus on the spine alone but should include a complete manual muscle examination and hip examination for asymmetric hip abduction contracture as surgical release of this may be all that is needed to allow the spine to straighten. Antero-posterior and lateral radiographs should be taken with the patient sitting unsupported.

### Management of children under 8 years of age

Children with early-onset scoliosis who have a severe curve are very difficult to manage. Spinal orthoses usually have no role to play in the care of these children. Spinal surgery is risky because of the severe pulmonary limitations, and especially if undertaken in facilities with limited resources. The use of ‘growing rods’ is fraught with complications in even the best of facilities.

### Management of children between 8 years of age and puberty

The spines of children <14 years from this second group tend to be much more flexible than those seen in idiopathic scoliosis. Thereafter, the curves tend to become rigid quickly. Consequently, an increasing curve seen on upright films, which would ordinarily signal the need for surgery in a child with idiopathic scoliosis, can often be ignored temporarily in younger children with poliomyelitis. The indication for surgery is not ‘progression alone’, but stiffening of a progressing curve noted on bending radiographs. For an example, an 8-year-old child with a curve which has progressed to 40° but which bends down to 20° may be seen to progress to 80°, over the next 4 years, while the bending film still shows the curve reducing to 20°. Surgery delayed until age 12 years, will result in a curve no worse than if it had been fused at age 8 years, yet the child will be taller and the need for extendable internal fixation with its concomitant complications can be avoided.

Children with paralytic scoliosis often use their curve, particularly the kyphotic and lordotic elements to balance their trunks. The great majority of children in non-Western countries sit on the floor. Following straightening of the spine surgically they are inclined to fall over if they sit on the floor. Fortunately, this is usually only temporary. Sitting in a chair corrects the problem but that solution may not be popular in a culture where socialization takes place at carpet level. The parents should be warned accordingly.

Adequate correction of lumbar scoliosis is necessary to correct the pelvic obliquity, leg length difference and the uncovering of the hip joint. It is very important to maintain lumbar lordosis if gluteus maximus muscles are weak so that the patient can lean backward sufficiently to allow gravity to extend the hips.

In severe cases, pre-operative correction using halo-gravity or halo-femoral traction may be helpful. Fusion techniques depend on the facilities available to the surgeon. Lumbar curves can be corrected well with anterior instrumentation reinforced with a secondary posterior fusion [34, 35]. If care is taken during surgery to adequately de-rotate the lumbar spine, the risk of increasing the kyphosis can be minimised. While currently, most surgeons use pedicle fixation in scoliosis surgery there has been limited experience in its use for poliomyelitis.

### Timing of scoliosis surgery in relation to lower extremity surgery

If an older, non-walking child has a severe scoliosis at first presentation, it is our practice to fuse the spine first, before performing the lower limb releases required for standing. This is based on the observation that getting such an older child up and walking after lower limb releases and bracing may take many months, during which time the curve is worsening. Furthermore, if the leg procedures are performed first, the long ‘learning-to-walk’ process then has to be redone after the post-operative recumbency from the spinal surgery.

## The scope of bracing in polio

Bracing is most frequently needed for the lower limb; very infrequently, bracing may be required to control spinal deformity. Bracing of the upper limb is seldom indicated.

Lower limb braces may be needed in the initial paralytic phase to prevent postural deformities. Simple thermoplastic above-knee posterior shells without knee or ankle joints will suffice.

### Bracing of the lower extremity in the phase of residual paralysis

There are a few general principles of bracing in polio. Firstly, an attempt must be made to leave as many joints as possible free (i.e., unlocked) as the energy consumption increases considerably when joints are locked [36, 37]. Secondly, the orthosis must be as light as possible to minimize energy expenditure and consequently light weight thermoplastic orthoses are preferred to traditional metal and leather orthoses [37]. Thirdly, because poliomyelitis results in pronounced muscle atrophy and reduced soft tissues over bony prominences that have low pressure tolerance, areas of contact of the orthosis and the limb should be as large as possible with good surface matching of the orthosis to the body segment. Lever arms should be designed to be as long as possible so the pressure is minimized, and the straps used to maintain contact between the orthosis and body segment, should be as large as practical. Fourthly, wherever possible, attempt to discard the orthosis by the time the child is skeletally mature by surgical methods of stabilization.

Once the child begins to walk the need for including knee and ankle joints must be considered. Since the child must be old enough to learn to lock the knee joint while standing and unlock it while sitting, including a knee joint in the orthosis may be deferred until the child is 5 years of age. The type of knee joint that is to be incorporated in the orthosis will vary with age, the need for bilateral bracing and the quadriceps power (Table 3).

**Table 3:** Factors that determine the type of joint to incorporate in traditional orthosis

Age of the child	Side requiring bracing	Quadriceps power	Type of knee joint to be used in orthosis
<5 years	Unilateral or bilateral brace	Grade III power or less	No knee joint
>5 years	Unilateral or bilateral	Grade III power	Posterior offset knee joint
>5 years	Unilateral	Less than Grade III power	Drop lock knee joint
>5 years	Bilateral	Less than Grade III power	Swiss knee joints (syn. bail lock)

The extent of bracing needed in the phase of residual paralysis depends on the muscle power around the hips, knees and ankles; if the quadriceps power is over grade 3, bracing need not extend proximal to the knee. An outline of indication for bracing of the lower limb in polio is shown in Table 4 [38].

**Table 4:** Indications for bracing of the lower limb in children in the phase of residual paralysis following polio

Indications	Aim	Orthosis
Quadriceps power Grade IV or V Foot drop No fixed deformity Tendon available for tendon transfer but child too young to co-operate with rehabilitation after transfer	Prevent foot drop during swing phase Overcome high-stepping gait Prevent rigid equinus from developing before the tendon transfer is performed	Thermoplastic ankle foot orthosis with trim lines posterior to malleoli—leaf spring orthosis (to be worn until tendon transfer is performed)
Quadriceps power Grade IV or V Dynamic equinovarus or equinovalgus (no fixed deformity) Tendon available for tendon transfer but child too young to co-operate with rehabilitation after transfer	Prevent rigid equinovarus or equinovalgus from developing before the tendon transfer is performed	Thermoplastic ankle foot orthosis with trim lines anterior to malleoli (to be worn until tendon transfer is performed and 6 months following tendon transfer)
Quadriceps power Grade IV or V Dynamic equinovarus or equinovalgus corrected by tendon transfer	To protect the transferred tendon from stretching and becoming ineffective	Thermoplastic ankle foot orthosis with trim lines anterior to malleoli (to be worn for 6 months following tendon transfer and then discarded)
Quadriceps power Grade III or less No flexion or recurvatum deformity at knee No rigid deformity of the foot and ankle Other knee normal	Prevent the knee from buckling during single leg stance Permit knee flexion during swing	Thermoplastic floor reaction orthosis^a^ (molded with ankle in 10° of plantarflexion)
Quadriceps power Grade III or less Recurvatum deformity at knee that is passively correctable No rigid deformity of the foot and ankle Other knee normal	Prevent the knee from buckling during single leg stance Prevent the recurvatum deformity from progressing Permit knee flexion during swing	Lehneis modification of the floor reaction orthosis (high popliteal trim line and suprapatellar extension)
Quadriceps power Grade III or less of both knees No deformity at knee No rigid deformity of the foot and ankle	Prevent the knees from buckling during single leg stance Permit knee flexion during swing on one side	Thermoplastic floor reaction orthosis on stronger limb and knee−ankle–foot orthosis with drop-lock knee joint on weaker limb
Power of hip muscles less than Grade III Quadriceps power Grade III or less No flexion deformity at knee No rigid deformity of the foot and ankle Other hip normal	Prevent hip instability Prevent the knee buckling during single leg stance	Knee−ankle–foot orthosis with thermoplastic ischial bearing quadrilateral socket, double irons and drop-lock knee joint
Power of muscles of both hips less than Grade III Quadriceps power Grade III or less No flexion deformity at knees No rigid deformity of the feet and ankles	Prevent instability of both hips Prevent the knees from buckling during single leg stance	Bilateral knee-ankle–foot orthosis with thermoplastic ischial bearing quadrilateral socket, double irons and Swiss knee joints (bail locks)

^a^Floor reaction orthosis (FRO) is also called ground reaction orthosis (GRO)

## Treatment of polio in resource limited areas

There are three areas where paediatric orthopaedic surgeons can help in a country with limited resources, namely identification, treatment and rehabilitation of children affected by polio. Identification of children with residual paralysis can be effectively carried out by conducting lameness surveys in local schools if school attendance is high; house-to-house surveys may be needed if the school attendance is poor [39–41].

While treating children with polio as visiting surgeons it is important that an effort be made to involve and train local surgeons with the long-term goal of capacity building to make them self-sufficient. Surgical techniques that are employed should be simple and inexpensive; a few examples are shown in Table 5. Wherever possible use inexpensive implants that can be implanted without the need for an image intensifier.

**Table 5:** Examples of simple options to treat flexion deformity of the knee in polio

Deformity	Recommended treatment	Advantages
Mild flexion deformity of the knee	Wedging of plaster casts	Low cost option as it avoids surgery and anaesthesia
Moderate flexion deformity of the knee	Spike osteotomy [42] and above-knee cast	Low cost option as it avoids the need for an implant for internal fixation
	Corrective osteotomy, crossed K-wire fixation and above-knee cast (avoid blade-plate and locked plate)	Low cost option as C-arm not required and implant is very cheap
Severe flexion deformity of the knee	Soft tissue release followed by gradual correction of the deformity by skeletal traction	Technically simple and low cost option as the need for a complex external fixator for gradual correction of the deformity is avoided

Rehabilitation is likely to be hampered by the lack of physiotherapeutic services and orthotic facilities. A physiotherapist should be included in the team visiting the country to teach the parent or care-giver of each child to provide the basic physiotherapy that may be needed in the post-operative period. Consider setting up an orthotic unit with the help of local artisans. Although low-cost orthotic appliances may be made from locally available material [43], thermoplastic orthoses may be better in the long run.

## Conclusions

Polio has not been eradicated and there is a risk of resurgence of the disease. Paediatric orthopaedic surgeons need to be prepared to deal with fresh cases of polio. Revival of old techniques of managing the effects of paralysis following polio is needed.
